# Comparative efficacy of Shatavari Ghrita eye drops with Shatavari Ksheerapaka versus Triphala Ghrita eye drops with Shatavari Ksheerapaka in postmenopausal ocular surface disease: a randomized, single-blind trial

**DOI:** 10.3389/fmed.2026.1824324

**Published:** 2026-07-22

**Authors:** B. N. Ashwini, K. Sivabalaji, Roshna Bhutada, Ramya Shruthi Janardhanan

**Affiliations:** 1 Department of Shalakya Tantra, Amrita School of Ayurveda, Amrita Vishwa Vidyapeetham, Amritapuri, India; 2 Department of Shalakya Tantra, Mahatama Gandhi Ayurveda College Hospital and Research Centre, Datta Meghe Institute of Higher Education and Research (DU), Wardha, Maharashtra, India; 3 Department of Shalakya Tantra, Ayurveda College, Coimbatore, India

**Keywords:** dry eye disease, OSDI, postmenopausal women, Shatavari Ghrita, Sushkakshipaka

## Abstract

**Introduction:**

Dry eye disease (DED) commonly affects postmenopausal women due to hormonal changes that disrupt tear film homeostasis. In Ayurveda, this stage corresponds to Vardhakya (aging), characterized by Vata predominance and Dhatu Kshaya (tissue depletion), making DED comparable to Shushkakshipaka, a Vata-dominant ocular disorder explained in Netra Roga. This study compared the efficacy of Shatavari Ghrita eye drops and Triphala Ghrita eye drops, each administered with systemic Shatavari Ksheerapaka for mild-to-moderate postmenopausal DED.

**Methods:**

The study was a randomized, single-blind, active-controlled superiority trial that included 120 postmenopausal women aged 45–65 years with clinically diagnosed mild-to-moderate DED. Participants were randomly allocated (1:1) into two groups (*n* = 60 each). Group A received Shatavari Ghrita eye drops (10 drops in both eyes) with oral Shatavari Ksheerapaka (100 mL twice daily before meals), while Group B received Triphala Ghrita (10 drops in both eyes) eye drops with the same systemic regimen. Treatment was administered for 30 days, followed by evaluations at the end of treatment and on days 45 and 60. Primary outcome measures included the Ocular Surface Disease Index (OSDI), Schirmer’s test I, and tear film break-up time (TBUT). Menopause-related quality of life was assessed using the Utian Quality of Life (UQOL) questionnaire. Statistical significance was set at a *p*-value of < 0.05, and between-group treatment effects were reported with 95% confidence intervals (CIs).

**Results:**

Both groups exhibited significant improvements from baseline in subjective symptoms and objective tear film parameters. Compared with Triphala Ghrita, Shatavari Ghrita produced significantly greater improvement in the OSDI (between-group *p* < 0.001), Schirmer’s test 1 (between-group mean difference 7.25 mm; 95% CI 6.39–8.11; *p* < 0.001), and TBUT (between-group mean difference 5.10 s; 95% CI 4.49–5.71; *p* < 0.001). No statistically significant between-group difference was observed in UQOL scores (*p* = 1.000).

**Conclusion:**

Shatavari Ghrita eye drops, administered with systemic Shatavari Ksheerapaka, were more effective than Triphala Ghrita eye drops in improving dry eye symptoms, tear secretion, and tear film stability in postmenopausal women with mild-to-moderate DED. These findings support Shatavari Ghrita as a promising Ayurvedic therapeutic option for the management of postmenopausal dry eye disease.

**Clinical Trial Registration:**

CTRI/2023/06/054213.

## Introduction

### Background and rationale

The ocular surface functions as an integrated functional unit, providing a smooth and stable refractive interface essential for optimal corneal optical performance (1). The morphology and function of the ocular surface are supported by a number of hormones, including sex steroids such as estrogen, thyroid hormone, prolactin, and growth hormone (2). Ocular pain, discomfort, and decreased visual acuity are symptoms of dry eye disease (DED), a multifactorial ocular surface condition. The incidence is particularly high in the menopausal and postmenopausal age groups. This is believed to be caused by shifts in the sex hormone balance occurring in these age groups of women. The production of the tear film’s aqueous layer, lipid, and mucin is influenced by sex hormones, specifically estrogens and androgens (3). The ocular surface expresses hormone receptors and is continuously exposed to circulating hormones (4). It is estimated that approximately 9.5% of women aged more than 75 years had DED, according to the Women’s Health Study, a sizable health survey carried out in North America (5). Using a similar methodology, the same study group (Schaumberg et al.) evaluated the prevalence of DED in men and found that women had a 70% higher prevalence of DED than 75 years. The same study group (Schaumberg et al.) assessed the prevalence of DED with a similar methodology and reported a 70% higher prevalence of DED among women than men in the 50 years and older age group. Hormone replacement therapy may be useful in treating menopause-related dry eye symptoms. Considering the gender predominance and the hormonal imbalance in women, it is important to find a better treatment alternative that addresses both the dry eyes and other symptoms related to menopause.

According to Ayurveda, Rajo Nivritti (cessation of menstruation) is a Swabhavika Vyadhi (natural physiological event). This phase is characterized by the predominance of Vata Dosha, along with Kapha Kshaya and progressive Dhatu Kshaya (tissue depletion) (6). The clinical features of postmenopausal dry eye disease closely resemble Shushkakshipaka, described under Sarvakshi Roga. Acharya Madhukosha defines Shushkakshipaka as ocular inflammation arising from excessive Shushkata (dryness) leading to Netra Upahata (damage to ocular structures) (7). Classical texts describe Vata as the principal dosha involved in its pathogenesis (8). Furthermore, Ashru (tear film) is considered the metabolic by-product of Rasa, Meda, and Asthi–Majja Dhatu (9, 10). Derangement of these Dhatu by the aggravated Vata impairs ocular lubrication and tear film stability, producing symptoms comparable to dry eye disease. Ayurvedic texts recommend Jeevaneeya (rejuvenative), Vatahara (Vata-alleviating), and Rasayana therapies to improve tissue strength and slow the degenerative changes. Local ocular treatments, including Aschyotana (eye drop), Tarpana (therapeutic retention of medicated ghee or scum of ghee over the eyes), and preparations containing Ghrita (Ghee), are advocated to enhance ocular lubrication and nourish the ocular tissues (11). Shatavari is a versatile female tonic and is a Nehra roga pathya. It is rich in phytoestrogens and helps to reduce the menopause-related symptoms and cures dry eye (12). It is Vayasthapana (retards depletion of bodily tissues) and Rasayana (nourishes the tissues). Contemporary studies suggest that its steroidal saponins—Shatavarins—exhibit phytoestrogenic activity through affinity for estrogen receptors and possess antioxidant and anti-inflammatory properties, which may contribute to alleviating menopausal symptoms (13). Hence, this study aimed to study the effect of Shatavari Ghrita eye drops and Shatavari Ksheerapaka in comparison with Triphala Ghrita eye drops and Shatavari Ksheerapaka in postmenopausal women.

### Objectives

The study was designed to evaluate integrated Ayurvedic treatment regimens comprising both systemic and topical interventions for postmenopausal ocular surface disease. Accordingly, Shatavari Ksheerapaka was administered uniformly to all participants as a common systemic intervention, while the topical treatments differed between the groups. This approach was based on the Ayurvedic understanding of Shushkakshipaka as a Bheshaja Sadhya Vyadhi (curable with medical intervention) requiring systemic as well as local management and enabled a comparison of the relative effectiveness of the two topical formulations within a standardized systemic treatment framework. Hence, systemic Shatavari Ksheerapaka was standardized across both groups to provide an identical background therapy. The topical eye drops were Shatavari Ghrita and Triphala Ghrita.

#### Primary objective

The primary objectives were as follows:

- To study the effect of Shatavari Ghrita eye drops and Shatavari Ksheerapaka systemically in the management of postmenopausal dry eyes using Schirmer’s test I, tear film break-up time (TBUT), and the Ocular Surface Disease Index (OSDI) questionnaire to assess the quality of life related to dry eye.
- To study the effect of Triphala Ghrita eye drops and Shatavari Ksheerapaka systemically in the management of postmenopausal dry eyes using Schirmer’s test I, tear film break-up time (TBUT), and the Ocular Surface Disease Index **(**OSDI) questionnaire to assess the quality of life related to dry eye.
- To compare the effectiveness between both the groups.

#### Secondary objective

The secondary objective was to assess the effect of the therapy on quality of life using the Utian Quality of Life in postmenopausal women.

## Methodology

IEC permission number and Date: MGACHRC/IEC/Oct-2022/581. Dated 7-10-2022.

CTRI registration number: CTRI/2023/06/054213 [registered on 20/6/2023].

*Ethics statement*: The studies involving humans were approved by Datta Meghe Institute of Medical Science, Wardha, IEC-MGACHRC-2022-AYUR-581.

### Trial design

This study was a randomized, single-blind superiority clinical trial. Sample size calculation (superiority trial formula) was performed using the standard deviation of Schirmer’s test I before treatment performed in a previous study, Clinical Evaluation of Yashtimadhu Daruharidra Eye Drops in the Management of Dry Eye Syndrome (14).

*Trial setting*: The study was conducted at Mahatma Gandhi Ayurveda College and Research Centre, Salod (H), Wardha.

### Eligibility criteria

*Inclusion criteria*: The study included female patients aged 45–65 years who had attained menopause, either spontaneously or surgically, and had clinical symptoms of dry eye syndrome with an OSDI score between 13 and 32 (mild to moderate), Schirmer’s test I between 5 and 10 mm (mild to moderate), and TBUT between 5 and 10 s (mild to moderate).

*Exclusion criteria*: The study excluded patients with any other ocular surface disease and dry eyes associated with syndromes or systemic disease. Patients on steroids and immunomodulatory/immunosuppressive therapy were also excluded (15).

### Intervention

Quality assurance of raw materials and medicine preparation was performed by a Good Manufacturing Practice (GMP)-certified company. Quality control (QC) tests of the prepared medicines were performed at the QC laboratory. Sterility tests of the Triphala Ghrita eye drop and Shatavari Ghrita eye drop were performed. Medicines were prepared according to the Ayurveda text Sharangadhara Samhita (16).

**Table 1:** 

Groups	Group A	Group B
No. of Subject	60	60
Drug	Shatavari Ghrita eye drops and Shatavari Ksheerapaka systemically.	Triphala Ghrita eye drops and Shatavari Ksheerapaka systemically.
Dose	10 drops of Shatavari Ghrita in both eyes. The frequency of administration—once daily, and retention period—100 Matra Kala. 100 ml Shatavari Ksheerapaka internally twice daily before food	10 drops of Triphala Ghrita in both eyes. The frequency of administration—once daily, retention period—100 Matra Kala. 100 mL Shatavari Ksheerapaka twice daily before food.
Duration of trial	30 days	30 days
Route of administration	Local and systemic	Local and systemic
Follow up	Two follow-ups, 45th and 60th day after the trial.	Two follow-ups, 45th and 60th day after the trial.

### Outcomes

**Figure 1:**
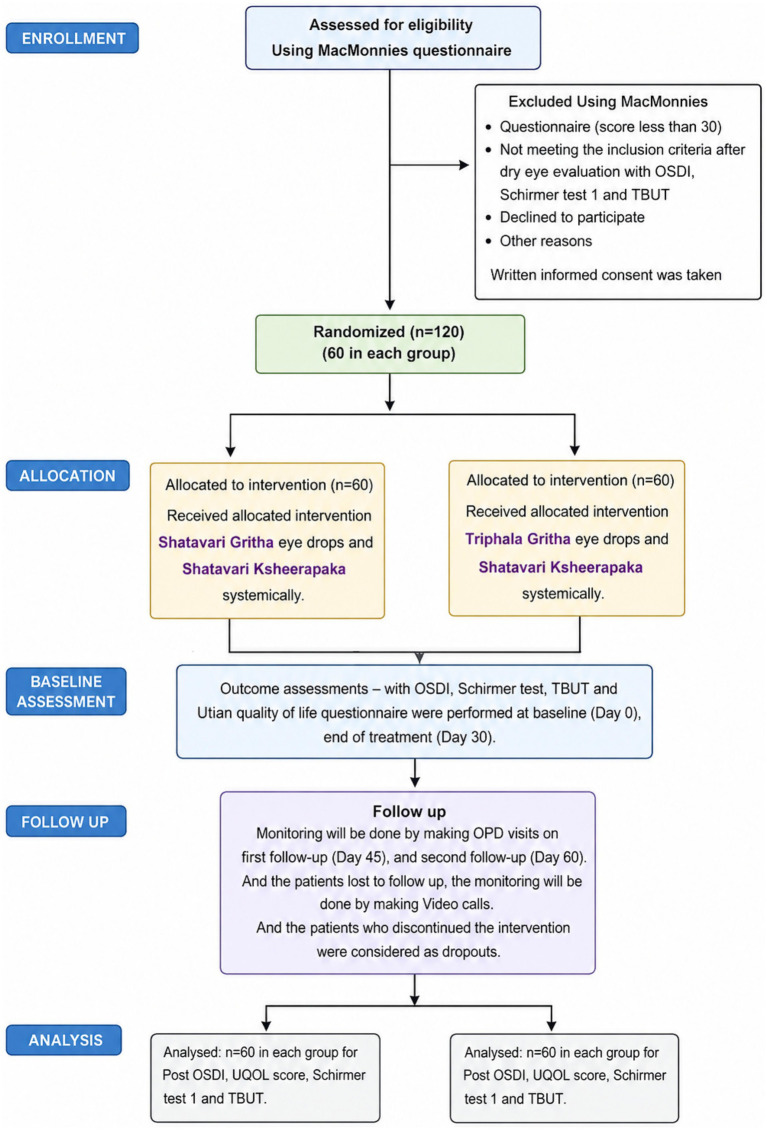
Consort flowchart.

Assessment was performed using objective parameters—Schirmer’s test I and tear film break-up time. Subjective assessments comprised the OSDI questionnaire to evaluate the quality of life related to dry eyes and the Utian Quality of Life questionnaire to assess the effect of the treatment on the quality of life of postmenopausal women. Outcome assessments were performed at baseline (Day 0), end of treatment (Day 30), first follow-up (Day 45), and second follow-up (Day 60). Outcome assessments were performed by the treating investigator (Ayurvedic ophthalmologist) using standardized procedures (see Figure 1).

### Sample size

The sample size was estimated *a priori* using a comparison of two independent means based on a pilot study. Sample size calculation has been revised to provide the complete basis for sample size estimation. The sample size was estimated *a priori* using a comparison of two independent means based on pilot study data, with Schirmer’s test I selected as the primary endpoint for sample size estimation. The calculation assumed a two-sided significance level of 5% (*α* = 0.05; *Z* = 1.96), 80% statistical power (*β* = 0.20; *Z* = 0.84), and an equal allocation ratio (1:1) between the two treatment groups. Based on the pilot study, the mean improvement in Schirmer’s test I in the control group was 9.18 mm (SD 4.372). Assuming a 20% clinically meaningful superiority of the experimental intervention, the expected mean improvement was 11.02 mm (SD 2.322). The sample size was calculated using the following formula for comparison of two independent means:


$$n=\frac{{\left(Z_{1-\alpha /2}+Z_{1-\beta }\right)}^{2}(\sigma_{1}^{2}+\sigma_{2}^{2}/r)}{{\left(\mu_{1}-\mu_{2}\right)}^{2}}$$

Where *μ*₁ and μ₂ represent the expected mean improvements in the control and experimental groups, respectively, *σ*₁ and σ₂ are their respective standard deviations, and r is the allocation ratio. The calculated minimum sample size was 55 participants per group. After accounting for an anticipated 10% dropout rate, the final sample size was increased to 60 participants per group, providing a total sample size of 120 participants.

### Randomization

Participants were randomly assigned to either intervention (Group A or Group B) using a lottery-based randomization method with a 1:1 allocation ratio. The allocation sequence was prepared by an independent individual not involved in participant recruitment, and the formal allocation concealment was not implemented.

### Blinding

This was a single-blind trial in which participants remained unaware of their treatment allocation throughout the study. Owing to the nature of the interventions, investigators responsible for treatment administration and outcome assessment were not blinded.

### Statistical methods

Statistical analysis was performed using SPSS software version 30.0.0.

The Wilcoxon signed-rank test (within-group analysis of the OSDI and the Utian Quality of Life): The Ocular Surface Disease Index (OSDI) and Utian Quality of Life (UQOL) scores are derived from questionnaire-based ordinal scales. Therefore, the Wilcoxon signed-rank test was used to compare paired observations (baseline, post-treatment, and follow-up) within each treatment group.

The paired *t*-test (within-group analysis of Schirmer’s test and tear film break-up time): Schirmer’s test I and TBUT were continuous quantitative variables measured in the same participants before and after treatment. As these variables were normally distributed, the paired *t*-test was used to compare the mean values within each treatment group.

The Mann–Whitney *U* test (between-group analysis of OSDI and UQOL): OSDI and UQOL scores were compared between the two independent treatment groups using the Mann–Whitney *U* test because these questionnaire scores were ordinal/non-parametric.

The unpaired (independent-samples) *t*-test (between-group analysis of Schirmer’s test I and tear film break-up time): Schirmer’s test I and TBUT values were compared between the Shatavari Ghrita and Triphala Ghrita groups using the unpaired (independent-samples) *t*-test because these outcomes were continuous variables measured in two independent groups, and the data met the assumptions for the parametric analysis.

## Results

### Within-group analysis of Shatavari Ghrita and Shatavari Ksheerapaka on subjective and objective parameters

#### Ocular Surface Disease Index (OSDI)

OSDI scores decreased significantly following treatment in the Shatavari Ghrita group, and the improvement was maintained in the Wilcoxon signed-rank test throughout the follow-up period (all *p* < 0.001).

#### Utian Quality of Life (UQOL)

The occupational, emotional, and sexual domains of the UQOL questionnaire showed significant improvements following treatment in the Shatavari Ghrita group. These improvements were sustained in the Wilcoxon signed-rank test during both follow-up visits (all *p* < 0.001).

#### Schirmer’s test I

Schirmer’s test I increased significantly in both eyes. In the right eye, the mean paired difference was 10.63 ± 2.62 mm with 95% CI: 9.96–11.31 mm; *t* = 31.40, *p* < 0.001. In the left eye, the corresponding mean paired difference was 10.80 ± 2.59 mm (95% CI: 10.13–11.47 mm; *t* = 32.39, *p* < 0.001). The improvements were maintained during both follow-up assessments.

#### Tear Film Break-Up Time (TBUT)

TBUT increased significantly following treatment in both eyes. In the right eye, the mean paired difference was 8.35 ± 1.86 s, with 95% CI of 7.87–8.83 s (*t* = 39.55, *p* < 0.001). In the left eye, the mean paired difference was 8.53 ± 1.63 s, with 95% CI of 8.11–8.95 s (*t* = 40.51, *p* < 0.001). These improvements remained significant during the follow-up period.

### Within-group analysis of Triphala Ghrita and Shatavari Ksheerapaka on subjective and objective parameters

#### Ocular Surface Disease Index (OSDI)

Within the Triphala Ghrita group, OSDI scores decreased significantly following treatment. The Wilcoxon signed-rank analysis exhibited a statistically significant reduction in OSDI scores from baseline to post-treatment, with the improvement maintained at both follow-up visits with a *p-value* of < 0.001.

#### Utian Quality of Life (UQOL)

The occupational, emotional, and sexual domains of the UQOL questionnaire showed significant improvements following treatment in the Triphala Ghrita group. These improvements were sustained during both follow-up visits in the Wilcoxon signed-rank test, (all *p* < 0.001).

#### Schirmer’s test I

Schirmer’s test I values increased significantly following treatment in both eyes. In the right eye, the mean paired difference between baseline and post-treatment was 3.38 ± 1.99 mm (95% CI: 2.87 to 3.90 mm), which was statistically significant (*t* = 13.15, *p* < 0.001). In the left eye, the mean paired difference was 4.23 ± 2.66 mm (95% CI: 3.55 to 4.92 mm), also exhibiting a statistically significant improvement (*t* = 12.34, *p* < 0.001). These improvements were maintained throughout the follow-up period.

#### Tear film break-up time (TBUT)

TBUT increased significantly following treatment in both eyes. In the right eye, the mean paired difference between baseline and post-treatment was 3.38 ± 1.99 s (95% CI: 2.87 to 3.90 s), with a statistically significant improvement (*t* = 13.15, *p* < 0.001). In the left eye, the mean paired difference was 3.58 ± 1.96 s (95% CI: 3.08 to 4.09 s), which was also statistically significant (*t* = 14.16, *p* < 0.001). The improvements were sustained during both follow-up assessments.

### Between-group analysis

Results on the comparison between Shatavari Ghrita eye drops with Shatavari Ksheerapaka and Triphala Ghrita with Shatavari Ksheerapaka on the ocular Surface Disease Index:

#### Ocular Surface Disease Index (OSDI)

Post-treatment OSDI scores differed significantly between the groups. The Shatavari Ghrita group exhibited significantly lower OSDI scores than the Triphala Ghrita group using the Mann–Whitney *U* test, with a *p*-value of < 0.001, indicating a greater reduction in dry eye symptoms. The observed percentage improvement was higher in the Shatavari Ghrita group.

#### Utian Quality of Life (UQOL)

No statistically significant difference in post-treatment UQOL scores was observed between the two treatment groups using the Mann–Whitney *U* test (*p* = 1.000), indicating that both interventions produced comparable improvements in quality of life.

#### Schirmer’s test I

Post-treatment Schirmer’s test I values were significantly higher in the Shatavari Ghrita group than in the Triphala Ghrita group. The between-group mean difference was 7.25 mm, with a 95% CI of 6.39–8.11 mm (independent-samples *t*-test, *p* < 0.001). The observed percentage improvement was greater in the Shatavari Ghrita group than in the Triphala Ghrita group.

#### Tear film break-up time (TBUT)

Post-treatment TBUT was significantly higher in the Shatavari Ghrita group than in the Triphala Ghrita group. The between-group mean difference was 5.10 s, with a 95% CI of 4.49–5.71 s (independent-samples *t*-test, *p* < 0.001), indicating greater improvement in tear film stability in the Shatavari Ghrita group.

## Discussion

Dry eye disease is more prevalent in women after menopause, and hormonal alterations have been implicated in its pathophysiology (17, 18). Experimental studies have identified sex hormone receptors in the lacrimal and meibomian glands (19), while clinical observations have reported associations between hormonal deficiency states and impaired tear production (20). Collectively, these findings support the role of sex hormones in maintaining ocular surface homeostasis and tear film integrity.

In this study, both Shatavari Ghrita and Triphala Ghrita administered with Shatavari Ksheerapaka produced statistically significant improvements in Schirmer’s test I, TBUT, OSDI, and UQOL scores from baseline. The mean improvement in Schirmer’s test I was 10.63 ± 2.62 mm and 10.80 ± 2.59 mm in the right and left eyes, respectively, in the Shatavari Ghrita group, compared with 3.38 ± 1.99 mm and 4.23 ± 2.66 mm in the Triphala Ghrita group. Similarly, the mean improvement in the TBUT was 8.35 ± 1.86 s and 8.53 ± 1.63 s in the Shatavari Ghrita group, compared with 3.38 ± 1.99 s and 3.58 ± 1.96 s in the Triphala Ghrita group. Compared with Triphala Ghrita, Shatavari Ghrita produced significantly greater improvement in the OSDI (between-group *p* < 0.001), Schirmer’s test I (between-group mean difference 7.25 mm; 95% CI 6.39–8.11; *p* < 0.001), and the TBUT (between-group mean difference 5.10 s; 95% CI 4.49–5.71; *p* < 0.001). No statistically significant between-group difference was observed in UQOL scores (*p* = 1.000).

Shatavari (*Asparagus racemosus*) is a classical Ayurvedic *Rasayana* widely used in women’s health. Phytochemical investigations have identified several constituents, including rutin, kaempferol, genistein, daidzein, and quercetin, that exhibit estrogen receptor-binding activity in experimental models (21). Clinical studies have also reported improvements in menopausal symptoms and quality of life following Shatavari supplementation (22). The better improvement observed in the Shatavari Ghrita eye drop group may be attributable to the aforesaid pharmacological properties of Shatavari; however, the underlying mechanism was not investigated in this study.

Both study formulations used ghee as the vehicle. Ghee contains fatty acids, antioxidant constituents, and fat-soluble vitamins (23, 24), which have been proposed to support ocular surface lubrication and epithelial integrity. Previous studies have suggested that dietary omega-3 fatty acids may improve tear film stability and reduce ocular surface inflammation (25). Both study groups additionally received Shatavari Ksheerapaka as systemic therapy. As Shatavari has traditionally been described as a *Rasayana* (26–28) and has been reported to improve menopausal symptoms in previous studies (29, 30), the improvements observed in quality-of-life outcomes may have been influenced by the systemic intervention received by both groups. Although greater improvements in Schirmer’s test I and TBUT were observed in the Shatavari Ghrita group, these findings should be interpreted within the context of the study design.

According to Ayurvedic principles, Shatavari possesses Madhura and Tikta Rasa, Guru and Snigdha Guna, Sheeta Veerya, and Madhura Vipaka. These properties are traditionally described as Vata Pitta Shamaka and are considered beneficial in the management of Shushkakshipaka, a condition characterized by aggravation of Vata dosha. Triphala, in contrast, is predominantly Kashaya Rasa. Classical Ayurvedic texts describe Kashaya Rasa as having Ruksha (drying) properties; therefore, this might have contributed to the reduced efficacy when compared to the Shatavari Ghrita eye drop group.

## Conclusion

Therefore, the findings of this study suggest that Shatavari Ghrita eye drops, when used in combination with Shatavari Ksheerapaka, may provide better clinical benefit than Triphala Ghrita eye drops for improving dry eye symptoms and tear film parameters in postmenopausal women. Further multicenter studies with longer follow-up and mechanistic investigations are warranted to confirm these findings.

### Limitations and future directions

This study was designed to evaluate comparative clinical efficacy rather than the underlying biological mechanisms. Hormonal profiling, estrogen receptor activity, ocular pharmacokinetics, lacrimal and meibomian gland function, tear film lipid composition, and ocular surface biomarkers were not assessed and should be included in future studies. The fatty acid composition of the ghee-based formulations was not analyzed in this study. Future investigations should characterize the formulation to determine the contribution of the ghee vehicle to the observed therapeutic effects. This was a single-center study with a relatively short follow-up period, which may limit the generalizability of the findings. Larger multicenter randomized controlled trials with extended follow-up are required to validate the long-term efficacy and safety of the interventions. Future studies should incorporate objective assessments such as meibomian gland imaging, tear film lipid analysis, ocular surface inflammatory biomarkers, and pharmacokinetic evaluations. These investigations would help elucidate the mechanisms of action of Shatavari Ghrita and strengthen the evidence for its use in postmenopausal dry eye disease.

## Data Availability

The original contributions presented in the study are included in the article/supplementary material, further inquiries can be directed to the corresponding author.
